# Dupilumab for cancer-associated refractory pruritus

**DOI:** 10.1016/j.jacig.2023.100128

**Published:** 2023-06-23

**Authors:** Aviv Talmon, Shlomo Elias, Limor Rubin, Yaarit Ribak, Eyal Ben Dori, Oded Shamriz, Michal Lotem, Irit Adini, Yuval Tal

**Affiliations:** a Allergy and Clinical Immunology Unit, Hadassah Medical Center, Faculty of Medicine, Hebrew University of Jerusalem, Jerusalem, Israel; b Department of Bone Marrow Transplantation and Cancer Immunotherapy, Hadassah Medical Center, Faculty of Medicine, Hebrew University of Jerusalem, Jerusalem, Israel; c Sharett Institute of Oncology, Hadassah Medical Center, Faculty of Medicine, Hebrew University of Jerusalem, Jerusalem, Israel; d Harvard Medical School, Department of Surgery, Center for Engineering in Medicine and Surgery, Massachusetts General Hospital, Boston, Mass

**Keywords:** Oncologic pruritus, chronic pruritus, itch, IL-4, IL-13, IL-31, dupilumab

## Abstract

**Background:**

Pruritus can be an intolerable symptom in patients with cancer. Type 2 inflammation, and specifically, the cytokines IL-4, IL-13, and IL-31, play major roles in the itching process. Dupilumab is an antibody against IL-4Rα, which is a common IL-4 and IL-13 receptor subunit. Blocking IL-4 and IL-13 activity reduces the synthesis of IL-31, the “itch cytokine,” and receptors for these 3 cytokines are expressed on itch nerves. Dupilumab is approved for treating moderate-to-severe atopic dermatitis, of which itching is a significant symptom.

**Objective:**

The objective of this case study was to present the initial evidence of the safety and efficacy of dupilumab as a treatment for intractable malignancy-associated pruritus in 3 patients, thereby providing a basis for further investigation in a larger cohort.

**Methods:**

As a proof of concept, we used dupilumab in our center to treat 3 patients with intractable malignancy-associated pruritus. The first patient was a 73-year-old male with a history of prostate cancer, the second patient was a 75-year-old female with cutaneous T-cell lymphoma, and the third patient was a 32-year-old male with metastatic melanoma. All 3 patients experienced debilitating itching, which started at some stage after the malignancy had been diagnosed. Moreover, none of the 3 patients showed clinical evidence of atopic dermatitis or other causes of itching (eg, uremia or liver failure), and none of the 3 patients responded to conventional treatments for pruritus.

**Results:**

Biweekly treatment with dupilumab led to an immediate improvement in itching, which subsided entirely after a few doses without any significant adverse effects.

**Conclusion:**

We propose that dupilumab is a safe and effective treatment for intractable malignancy-associated pruritus, and we are currently testing it in a large cohort.

## Introduction

Chronic pruritus is defined as an itching sensation persisting for more than 6 weeks.^1^ Pruritis can impair sleep quality and work productivity, leading to depression and suicide ideation. Many disorders, both cutaneous (eg, atopic dermatitis, psoriasis) and noncutaneous (eg, chronic kidney disease, cholestasis), can cause pruritus.^1^ Pruritus is a substantial symptom of hematologic malignancies,^1^^,^^2^ and to a lesser extent, solid tumors.^1^^,^^3^

Many types of immune cells and mediators participate in the physiology of itching secondary to an inflammatory process, especially T_H_2 cells and mast cells. Consistent with a type 2 response, IL-4, IL-13, and IL-31 are major players at various points along the neural pathway in the itching cascade. IL-31, known as “the itch cytokine,” is the most prominent in the itch propagation cascade.^4^ Activated CD4^+^ (T_H_2) lymphocytes are a primary source of IL-31 secretion^5^; however, other immune cells may also be involved (eg, mast cells and eosinophils).^6^ A recent study showed that deletion of IL-4Rα (a subunit of both IL-4 and IL-13 receptors) reduced inflammation and itch sensation in mice.^7^ Receptors for all of the aforementioned cytokines reside along itch neurons in rodents and humans.^7^ We therefore postulate that blocking these receptors can alleviate cancer-related pruritus by directly affecting neurons, even if they are unrelated to T_H_2 cell inflammation.

Dupilumab is an antibody against IL-4Rα that is approved for several indications, including asthma,^8^ chronic rhinosinusitis with nasal polyposis,^9^ and atopic dermatitis,^10^ for which itching is one of the main symptoms. We hypothesize that dupilumab might be helpful in treating malignancy-associated pruritus, which could be mediated either by the disease itself or by its treatment. Here we describe 3 patients with refractory malignancy-associated pruritus without a rash who were successfully treated with dupilumab. All participants in this study, which was conducted under the protocol number 0557-22-HMO, provided informed consent before their involvement.

## Results and discussion

Patient 1 is a 73-year-old male with a medical history of prostate cancer who underwent a radical prostatectomy in March of 2018 followed by radiation therapy. He also received short-term hormonal therapy with triptorelin. The patient was referred to our immunology clinic in 2019 for persistent, diffuse itching, which began after his malignancy was diagnosed and before initiation of triptorelin treatment. The itching significantly interfered with his quality of life. On physical examination, the patient’s skin showed signs of excoriation with bleeding lacerations, especially on the back and arms, as well as a few areas of lichenification without any accompanying dermatoses (Peak Pruritus Numeric Rating Scale score^11^ [NRS] of 7). No eosinophilia or basophilia was noted in the blood test results. The patient was treated with topical tacrolimus and moisturizer, with mild improvement. Subsequently, the patient started receiving subcutaneous treatment with dupilumab every 2 weeks, and he is currently continuing with the treatment. The patient experienced marked improvement of his itching following the first injection, and after the third injection, the itching completely resolved (NRS score of 0). At the time of writing of this review, the patient had been undergoing treatment for a duration of 2 years and 3 months.

Patient 2 is a 75-year-old female who in February of 2006 was diagnosed with cutaneous T-cell lymphoma (CTCL) in which pruritus was a presenting sign. The patient was initially treated with whole-body electron beam therapy with a good response, but she eventually relapsed. She received several additional lines of treatment, including IFN-α, topical nitrogen mustard, and topical corticosteroids. By 2007, the patient’s lymphoma had metastasized to the lungs and she was treated with belinostat, gemcitabine, local retinoids, and eventually liposomal doxorubicin. In 2017, the patient developed a malignant melanoma *in situ* on her cheek, which was treated by local excision. The patients had experienced an intractable, excoriating itch since the diagnosis of CTCL. She was referred to our clinic in 2019 because the itching had worsened and was unresponsive to antihistamines, topical ointments, and systemic corticosteroids. On physical examination, the patient had mild diffuse erythema, which was incompatible with atopic dermatitis or urticaria. She also had signs of excoriation and skin lichenification secondary to chronic scratching (NRS score of 10). There was no eosinophilia or basophilia noted in her blood test results. She began receiving biweekly treatment with subcutaneous dupilumab in June 2019, and it is still ongoing. The itching improved significantly after the first dose and disappeared entirely after the third dose (NRS score of 0). As of the time of writing of this review, the patient had been undergoing treatment for a duration of 3 years and 5 months.

Patient 3 is a 32-year-old male who was diagnosed with melanoma in April 2019 after the appearance of a lesion on his shin. In July 2019, the patient received immunotherapeutic treatment with nivolumab. By April 2021, the patient was found to have melanoma metastases in the terminal ileum, for which he was treated with segmental resection of the small intestine. In June 2021, the patient began undergoing combination therapy with nivolumab and ipilimumab. He also received a tailored vaccine derived from his autologous tumor cells. During his disease course, the patient developed extensive excoriating itching, which significantly impaired his quality of life. The itching appeared after initiation of nivolumab-ipilimumab combination therapy and is therefore presumed to be related to the therapy. The patient was referred to our immunology clinic in April 2021. The itching worsened with showering, sexual intercourse, and physical activity. On physical examination, there were signs of excoriation but no evidence of rash; his NRS score was 9. The itching sensation did not improve after treatment with antihistamines or corticosteroids. Biweekly subcutaneous dupilumab was initiated and led to an overwhelming improvement and almost complete cessation of itch (NRS score of 2). Because of technical regulations, the patient received only 5 doses of dupilumab in total. Unfortunately, following the discontinuation of dupilumab therapy, the patient experienced recurrence of his pruritic symptoms.

**Fig 1:**
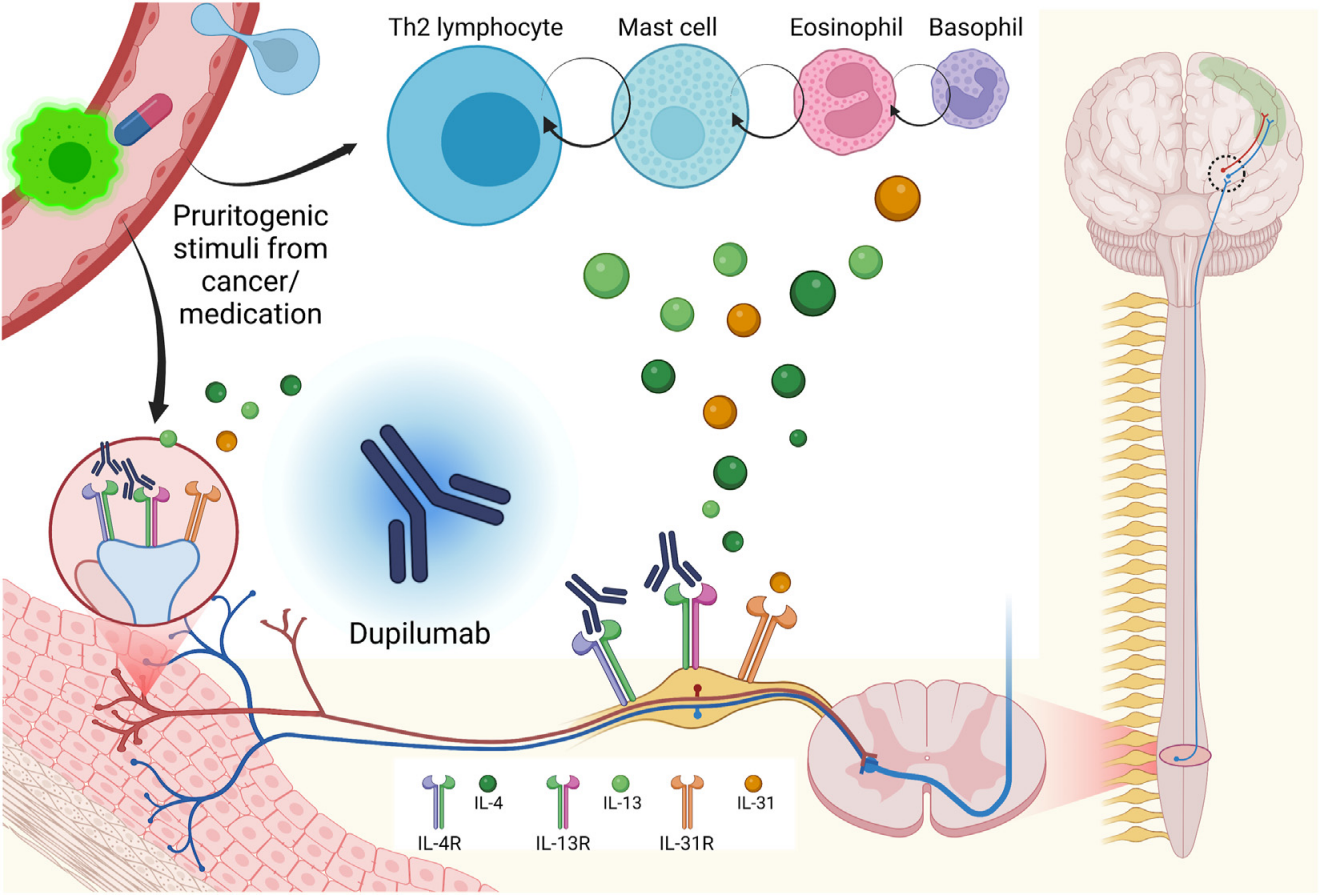
Illustration of the presumed mechanism of action of dupilumab in cancer-associated pruritus. In addition to suppressing of a T_H_2 cell response, dupilumab may directly interact with cytokine receptors on pruritic nerves, subsequently relieving pruritus.

Here we have reported the successful use of dupilumab for malignancy-associated pruritus. All 3 patients had a concurrent malignancy and experienced debilitating intractable itching that commenced after the malignancy diagnosis. None of the patients had clinical evidence of atopic dermatitis or other causes of itching unrelated to malignancy (eg, uremia). The blood eosinophil and basophil counts of all 3 patients were within normal limits. Their pruritus could be related to the malignancy or its treatment (as might be the case in the third patient, who was treated with checkpoint inhibitors). Importantly, all 3 patients taking dupilumab experienced marked improvement of their symptoms without any significant adverse effects. Although dupilumab does not directly block IL-31 (the main itch cytokine), IL-31 has been associated with the secretion of IL-4 and IL-13, which are directly affected by dupilumab.^12^ A study has shown that addition of anti–IL-4 antibody can significantly reduce IL-31 in T_H_2 cells *in vitro*, underscoring the necessity of IL-4 in IL-31 production.^13^ Furthermore, dupilumab has been found to reduce the expression of IL-31 in the skin of patients with atopic dermatitis.^14^ However, further research is needed to fully evaluate the impact of this treatment on the regulation of cytokine receptors on pruritic nerves. It is plausible that IL-4 and IL-13 are also implicated in pruritus because receptors for all 3 cytokines (IL-4, IL-13, and IL-31) are present along the itch neurons^7^ (Fig 1) and all 3 cytokines are involved in the mechanism of pruritus in CTCL.^15^ In contrast to the few previous reports, we have not noted progression of CTCL after the start of dupilumab.^16^ It should be emphasized that dupilumab alleviated pruritus in patients who did not have high levels of eosinophils or basophils, which are common features of type 2 inflammation. Therefore, we presume that the influence of the treatment on the neuronal itch pathway might be independent of the type of inflammation.

Despite these encouraging clinical observations, the efficiency of dupilumab for treating cancer-associated pruritus should be tested in a large randomized clinical trial. In addition, there are still several unanswered questions that require further investigation. Which patients with cancer who are experiencing pruritus could benefit from treatment with dupilumab and whether this treatment could be effective for treating pruritus caused by nonmalignant conditions that are not currently indicated for this medication are unclear. The ideal treatment duration is also uncertain, particularly in light of the symptom relapse experienced by patient 3 after treatment discontinuation. Furthermore, the mechanism of action of dupilumab for treating cancer-associated pruritus should be explored, possibly through methods such as immunohistochemistry. Additionally, assessing immune markers obtained from peripheral blood or biopsy samples could potentially serve as prognostic indicators for treatment response and duration in patients. The findings observed in this case series are currently being tested in a large prospective randomized control clinical trial, which will allow confirmation of the results of our observation in a large cohort and answer the aforementioned questions.^17^ This could pave the way for use of dupilumab in treating intractable cancer-associated pruritus.

## Disclosure statement

Disclosure of potential conflict of interest: A. Talmon, L. Rubin, and Y. Tal report having provided consulting services (including external delivery of lectures and service on medical boards) to several pharmaceutical companies, including Sanofi, Takeda, AstraZeneca, and Pfizer. It is important to note that this proof-of-concept trial did not receive external funding. I. Adani was awarded the Pathways Global Innovation Grant (which is not relevant to the current research) from Regeneron-Sanofi. The rest of the authors declare that they have no relevant conflicts of interest.
